# Adalimumab-Induced Pyodermatitis-Pyostomatitis Vegetans (PPV) in Ulcerative Colitis: A Rare Dermatological Dilemma

**DOI:** 10.7759/cureus.109534

**Published:** 2026-05-24

**Authors:** Fatima Belabbes, Zineb Basri, Madiha El Jazouly, Najat Lamalmi, Imane Ben El Barhdadi

**Affiliations:** 1 Department of Gastroenterology and Hepatology, Cheikh Khalifa International University Hospital, Mohammed VI University of Health Sciences (UM6SS), Casablanca, MAR; 2 Department of Dermatology, Cheikh Khalifa International University Hospital, Mohammed VI University of Health Sciences (UM6SS), Casablanca, MAR; 3 Department of Anatomical Pathology, Cheikh Khalifa International University Hospital, Mohammed VI University of Health Sciences (UM6SS), Casablanca, MAR

**Keywords:** adalimumab, anti-tnf therapies, dermatological manifestations, pyostomatitis vegetans, ulcerative colitis

## Abstract

Ulcerative colitis (UC), a chronic inflammatory disease affecting the rectum and colon, is commonly managed using immunosuppressants and biologics, including adalimumab. However, adalimumab administration can lead to dermatological complications, notably pyostomatitis vegetans, in UC patients. This study presents a unique case of pyostomatitis vegetans that developed in a UC patient following initiation of adalimumab therapy.

The primary challenge lies in accurately differentiating between adalimumab-induced pyostomatitis vegetans and the dermatological manifestations associated with UC. Precise identification of the origin of pyostomatitis vegetans is crucial for appropriate clinical intervention.

We report an exceptional case involving an 82-year-old female patient who presented with rectal discharge and a deteriorating overall condition. She had a prior diagnosis of UC and subsequently developed pyostomatitis vegetans after commencing adalimumab therapy. Treatment with adalimumab was discontinued in favor of topical corticosteroid administration, resulting in favorable outcomes. This study emphasizes the rarity of pyostomatitis vegetans, particularly in the context of adalimumab usage, underscoring the critical need for heightened vigilance in the clinical management of such occurrences.

## Introduction

Ulcerative colitis (UC) is a chronic inflammatory bowel disease (IBD) affecting the rectum and colon, characterized by epithelial barrier disruption, mucosal ulcerations, and lymphoplasmacytic infiltration of the lamina propria. UC frequently presents with extraintestinal manifestations, among which dermatological and mucocutaneous conditions are prominent. These include pyoderma gangrenosum (PG), erythema nodosum, and rarer forms such as pyodermatitis-pyostomatitis vegetans (PPV) [1].

The pathophysiology of IBD involves a dysregulated immune response to gut microbiota in genetically susceptible individuals. UC is classically associated with a Th2-predominant immune response, whereas Crohn's disease involves Th1 and Th17 pathways. During UC flares, the inflammatory burden increases systemically, often triggering or worsening extraintestinal manifestations.

PPV is a rare mucocutaneous condition often associated with IBD, particularly UC. It comprises two components: pyodermatitis vegetans: vegetating pustular lesions affecting the skin folds, scalp, head, and neck; pyostomatitis vegetans: snail‑track erosions and pustules on the oral mucosa (buccal, gingival, and palatine surfaces).

PPV is an exceptionally rare condition, with fewer than 100 cases reported in the literature to date. It is strongly associated with IBD, particularly UC, in more than 70% of reported cases. The mean age of affected patients is approximately 40 years, with a slight male predominance. Our patient, in her early 80s, is therefore atypical compared to the usual epidemiological profile.

In this case, it remains unclear whether PPV represents a severe extraintestinal manifestation of an underlying UC flare or a paradoxical reaction induced by recently initiated adalimumab therapy. We therefore propose two hypotheses to guide clinicians facing similar diagnostic challenges.

PPV is considered a variant of pyoderma gangrenosum within the spectrum of neutrophilic dermatoses and may share features with Sweet's syndrome and aseptic abscesses. Histologically, PPV lesions are characterized by intense neutrophilic and eosinophilic infiltration of the dermis and oral mucosa, without true vasculitis [2]. This distinguishes PPV from the primary intestinal lesions of UC, which show epithelial disruption and lymphoplasmacytic infiltration rather than neutrophilic predominance.

Adalimumab, a tumor necrosis factor-α (TNF-α) inhibitor, is commonly used to treat various chronic inflammatory diseases, including UC, due to its demonstrated efficacy in reducing inflammation [3]. However, anti-TNF therapy has been associated with paradoxical inflammatory reactions, including new‑onset or exacerbation of neutrophilic dermatoses such as PPV.

The relationship between adalimumab and mucocutaneous manifestations, particularly PPV, has garnered increasing interest within the medical community. To the best of our knowledge, this represents the first published case of its kind in Morocco. This exceptional case underscores the diagnostic challenge of distinguishing a mucocutaneous manifestation inherently linked to UC from one that may arise as a paradoxical reaction to adalimumab treatment. The ability to make this distinction is of paramount importance for clinical management, guiding therapeutic decisions, and determining appropriate medical interventions.

This study aims to contribute to our comprehension of the intricate interactions between adalimumab and mucocutaneous manifestations, specifically PPV. Additionally, it provides valuable insights to enhance the clinical management of patients with chronic inflammatory diseases, such as UC, who require adalimumab treatment. This report presents a unique case that highlights the clinical challenge of distinguishing whether PPV represents a severe extraintestinal manifestation of the underlying UC flare or a paradoxical reaction induced by recently initiated adalimumab therapy [4].

## Case presentation

A female patient in her early 80s presented with rectal bleeding and an altered general condition. She had no known history of digestive or extradigestive neoplasia, systemic diseases, hematological disorders, or other comorbidities. She had undergone a thyroidectomy 15 years ago and is currently taking levothyroxine.

She had been diagnosed with left-sided UC four years ago, with the diagnosis based on clinical, biological, endoscopic, and histological criteria. Her first colonoscopy revealed ulcerated subacute congestive rectocolitis, consistent with UC, with no prior extraintestinal manifestations. Her treatment regimen included mesalamine at a dose of 3 g (administered as 500 mg tablets) with two tablets taken three times a day, which she had been on since her diagnosis in 2020.

In 2023, the patient experienced a severe relapse necessitating oral corticosteroid therapy at a dose of 1 mg/kg/day (40 mg/day), with a tapering regimen as initial treatment. Azathioprine was introduced as background therapy at a dose of 2.5 mg/kg/day. Azathioprine 2.5 mg/kg was switched to mercaptopurine due to uncontrollable vomiting.

Despite the medication change, the patient continued to experience vomiting, raising suspicion of intolerance to immunosuppressants. This justified initiating biologic therapy with adalimumab (Amgevita). The patient received adalimumab for two months according to the standard dosing regimen: an initial dose of 80 mg at Week 0 (Day 1), then 40 mg at Week 2, and thereafter 40 mg every two weeks. Four days after the last dose, the patient began to experience bloody phlegm emissions, with up to eight bloody stools accompanied by mucus per day. Additionally, there was progressive bilateral swelling of the eyes and the development of reddish nodular skin lesions on the leg, the anterior cervical region, and the left armpit. Her condition had deteriorated further, with a fever of 39°C and a weight loss of 7 kg over two months.

Upon clinical examination at admission, the patient exhibited a performance status of 3-4, along with signs of skin dehydration and nonpalpable lymph nodes. Abdominal examination revealed diffuse tenderness with no palpable masses. A proctological examination revealed a deep posterior anal fissure and purplish nodular skin lesions, as well as bilateral and symmetrical pustules. Dermatological examination revealed purplish nodular lesions with bilateral and symmetrical pustules. A pustular lesion was also present in the left axillary fold (Figure 1A). Ophthalmological examination revealed extensive palpebral edema infiltrated with purulent crusts and swelling in both eyes (Figure 1B). The palpebral involvement was consistent with PPV-related mucocutaneous lesions and not with primary blepharitis. A nodular lesion was also present on the neck (Figure 1C). Ear, Nose, and Throat (ENT) examination revealed hemorrhagic vesicles with purulent crusts in both external auditory canals with otorrhea (Figure 1D). Oral examination revealed characteristic snail-track erosions involving the buccal mucosa. No photographs of the oral cavity were available.

**Figure 1:**
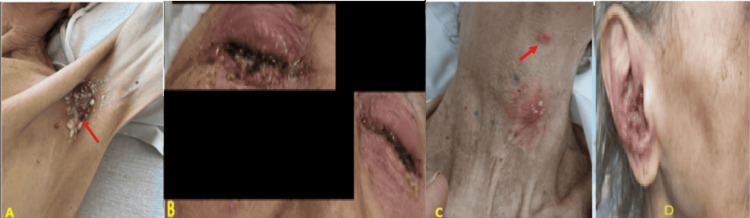
Photographs before treatment (A) Left axillary fold with pustules. Red arrows indicate pustules. (B) Palpebral edema with purulent crusts. (C) Nodular lesion on the neck. The red arrow indicates the lesion. (D) Hemorrhagic vesicles with purulent crusts in the external auditory canal with otorrhea

A thoracic-abdominal-pelvic CT scan revealed colonic thickening in stages, with no significant deep abdominal collection (Figure 2).

**Figure 2:**
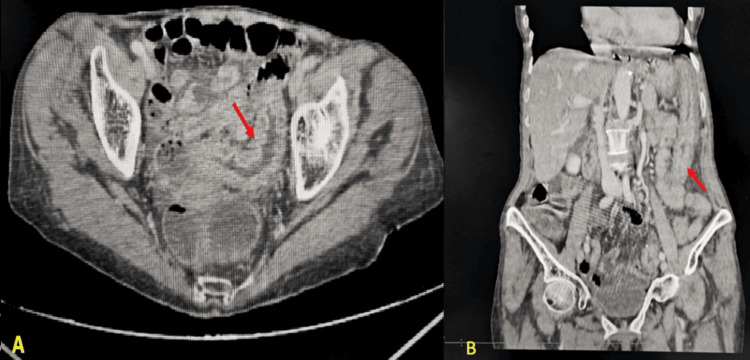
Abdominopelvic CT (A) Axial section: sigmoid wall thickening. (B) Coronal section: left colonic wall thickening

Biological investigations revealed elevated inflammatory markers, microcytic hypochromic anemia, hemoglobin level of 9.7 (11.8-16), elevated C-reactive protein at 155 (<6), erythrocyte sedimentation rate 60 (0-20), ferritin at 218 (20-400), hypokalemia at 2.8 (3.5-5.5), and a positive procalcitonin result. Viral serologies (HIV, hepatitis B virus, herpes simplex virus, cytomegalovirus) were negative (Table 1).

**Table 1:** Biological results CRP: C-reactive protein; HBV: hepatitis B virus; HSV: herpes simplex virus; CMV: cytomegalovirus

Parameter	Patient's value	Normal range
Hemoglobin	9.7 g/dL	11.8-16.0 g/dL
CRP	155 mg/L	<6 mg/L
Erythrocyte sedimentation rate	60 mm/hour	0-20 mm/hour
Ferritin	218 μg/L	20-400 μg/L
Potassium	2.8 mmol/L	3.5-5.5 mmol/L
Procalcitonin	1.25 μg/L	<0.25 μg/L
HIV serology	Negative	Negative
HBV serology	Negative	Negative
HSV serology	Negative	Negative
CMV serology	Negative	Negative

Rectosigmoidoscopy without insufflation and without careful colonic preparation showed a fragile, erythematous mucosa that bled easily, with map-like ulcerations and an altered vascular network, suggesting severe relapse of UC according to the Mayo score of 3.

The patient received antibiotic therapy with metronidazole (1.5 g/day) and ceftriaxone (2 g/day), combined with acyclovir (10 mg/kg/day) for three days, until a negative herpetic viral load was obtained. This was considered due to the purulent ENT and ophthalmological manifestations, fever, and elevated procalcitonin. A rectal enema consisting of Pentasa and corticosteroids was also administered to relieve intestinal symptoms. A skin biopsy of the cutaneous lesion confirmed PPV (Figure 3), and local treatments included topical corticosteroids.

**Figure 3:**
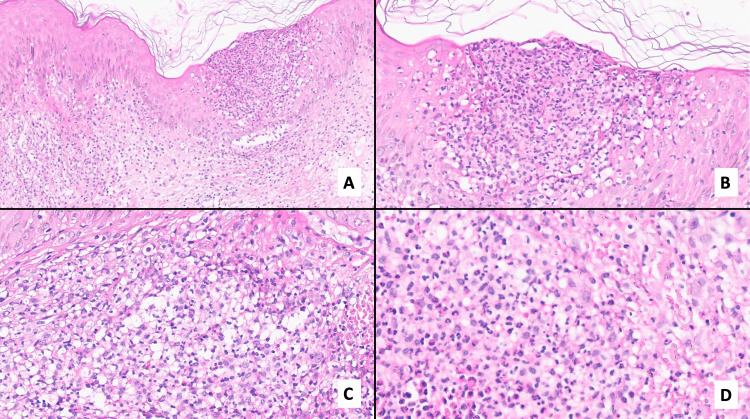
Neutrophilic pyoderma (H&E staining) (A) Low-power view (×40) showing an epidermal pustule associated with a dermal inflammatory infiltrate. (B) At medium magnification (×200), the epidermis appears spongiotic and scattered with predominantly intraepidermal neutrophilic pustules. (C,D) Under high magnification (×400), the dermal infiltrate consists mainly of intact and degenerated eosinophils and neutrophils within an edematous stroma, without identifiable microorganisms, findings consistent with a neutrophilic dermatosis of the neutrophilic pyoderma type H&E: hematoxylin and eosin

Ophthalmological and ENT lesions were managed with antibiotic and corticosteroid eye drops, along with daily local care. The patient exhibited significant improvement, with the disappearance of rectal symptoms, drying of skin lesions, reduction of palpebral edema, and resolution of ENT symptoms (Figure 4). Biological inflammatory markers also showed improvement.

**Figure 4:**
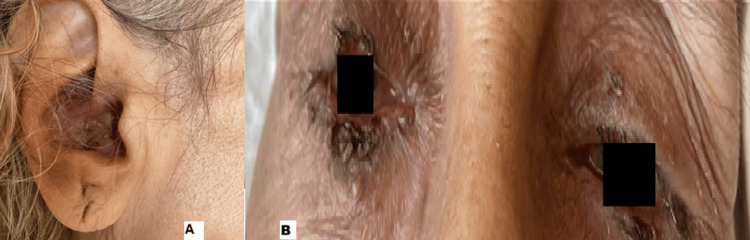
Posttreatment evolution (A) Drying of the otorrhea and crusted lesions in the external auditory canal. (B) Marked improvement in palpebral edema and ocular secretions

Regarding UC treatment, ustekinumab was proposed as an alternative biologic therapy given its mechanism of action targeting IL‑12/23, which may be effective in neutrophilic dermatoses. However, the patient declined this option for personal reasons, so a mesalamine-based treatment was maintained with a mesalamine enema. This resulted in good clinical and biological progress over a three-month period.

## Discussion

Dermatological extraintestinal manifestations are frequently observed in IBD, occurring in approximately 15% of cases with female predominance [5]. The most prevalent dermatological manifestations associated with IBD include erythema nodosum, followed by neutrophilic dermatoses such as PG and Sweet's syndrome. Additionally, rarer forms such as PPV and aseptic abscesses have also been reported [5].

PPV, initially described in 1949, is a rare inflammatory stomatitis often observed in association with IBD. It is more commonly seen in patients with UC than in those with Crohn's disease. The recognition of PPV is growing, and it is sometimes mistakenly attributed to the underlying causative disease, underscoring the importance of our discussion. In this case, we propose two hypotheses to explain the onset of PPV in this UC patient.

Hypothesis 1

Hypothesis 1 is a direct association between the onset of PPV and the exacerbation of the IBD flare-up, particularly when the disease is classified as severe, as indicated by a Lichtiger score greater than 10. However, these manifestations tend to be more frequent in patients aged 25-50 years, which contrasts with the advanced age of our patient (early 80s). For instance, a case was reported involving a 26-year-old woman with UC who developed PPV two years after her UC diagnosis [2].

Another case reported by Baeke et al. involves a 47-year-old woman treated for both UC and ankylosing spondylitis who also presented with PPV, although the timeframe of onset was not specified. She underwent two months of oral corticosteroid therapy combined with dapsone, resulting in complete remission [6]. Other authors, such as Mijandrusić-Sincić et al. [7], reported two additional similar cases.

Hypothesis 2

Hypothesis 2 is a drug-induced origin. Very few cases of post-adalimumab PPV have been documented. For instance, Baeke et al. reported a case involving a 31-year-old woman undergoing adalimumab treatment for ankylosing spondylitis who presented with PPV localized in the mouth, genital area, and folds. Her condition improved significantly on dapsone therapy given her corticosteroid dependency [6].

From a pathophysiological perspective, UC is a chronic inflammatory disease characterized by epithelial lesions resulting in ulcerations, coupled with lymphoplasmacytic infiltration of the lamina propria. At the cellular level, there is dysregulation of lymphocyte activity, marked by overexpression of Th2 at the expense of Th1 and Th17, a phenomenon further amplified by the Nuclear Factor kappa-light-chain-enhancer of activated B cell pathway. Th2 helper lymphocytes stimulate secretion of various cytokines, including TNF‑α [8].

Treatments such as adalimumab, an anti‑TNF‑α monoclonal antibody, suppress inflammatory factors by modulating immune activity through induction of T‑cell apoptosis in the lamina propria. This effect is more pronounced in lamina propria T lymphocytes than in circulating T lymphocytes and is mediated by caspase‑3 activation [9]. For this reason, adalimumab has emerged as a cornerstone in the management of UC, particularly for extraintestinal manifestations such as PPV.

Immunohistochemistry has identified significant eosinophilic and neutrophilic infiltration in PPV skin lesions, accompanied by overexpression of interleukin‑36, leading to paradoxical inflammation in IBD patients on anti‑TNF‑α [10,11]. Other studies have shown abnormal overexpression of interleukins 17 and 22 in psoriatic lesions during anti‑TNF‑α administration, attributed to Th1 cells [12]. It is plausible that this alteration in the cytokine profile could be associated with specific blockade of certain cytokines by biologic therapy, prompting the question of a causal link between adalimumab and PPV in UC patients.

Lesions associated with PPV typically manifest as snail‑track erosions on an erythematous background. They are found primarily in the oral, ocular, and auditory orifices but may also appear in skin folds [4]. The diagnosis of PPV is based on clinical presentation and the exclusion of other skin conditions, confirmed by histology. These lesions typically appear on the head, neck, trunk, and upper limbs [4].

In our patient, lesions were nodular, erythematous, tense, and occasionally pustular and vesicular, notably in the auditory and ocular canals. Histology ruled out other dermatoses and established PPV. Standard management would involve corticosteroids or immunosuppressants, but since the latter had already commenced, this side effect may be treatment‑related [12].

Clinically, distinguishing neutrophilic dermatoses is challenging. PG encompasses several variants [1]: ulcerative PG (sterile pustules evolving into necrotic ulcers) [13]; pustular PG (multiple painful pustules, often associated with UC, may mimic Sweet's syndrome); bullous PG (differential diagnosis for atypical Sweet's syndrome) [13]; and vegetative PG (chronic nodular form, may mimic aseptic abscesses).

PPV is a probable variant of pustular PG with buccal localization (snail‑track erosions). About half of PPV cases also involve skin (axillary/inguinal folds, scalp), resembling our patient's presentation [4].

Treatment for PPV follows that of neutrophilic dermatoses: first‑line: topical corticosteroids, systemic prednisone (0.5 mg/kg/day), colchicine, or dapsone [4]; second‑line: immunosuppressants (azathioprine, methotrexate). If side effects occur, cyclosporine or cyclophosphamide may be considered [4]. Biotherapies (infliximab, adalimumab, ustekinumab) are reserved for systemic disorders associated with IBD or renal disease. Ustekinumab may be considered when neutrophilic dermatosis arises during anti‑TNF therapy, given its IL‑12/23 mechanism [12].

Limitations

Oral photographs were not obtained at the time of presentation. However, clinical examination confirmed snail‑track erosions, establishing pyostomatitis vegetans.

## Conclusions

PPV in the context of chronic IBD, specifically UC, remains a rare and intricate condition. A review of the literature identified fewer than 100 reported cases, with a mean age of approximately 40 years and a slight male predominance. Our patient, a female in her early 80s, is therefore atypical compared to the usual epidemiological profile.

This case highlights the diagnostic dilemma of distinguishing whether PPV represents a severe extraintestinal manifestation of an underlying UC flare or a paradoxical reaction induced by adalimumab. Our data demonstrate a temporal association between adalimumab initiation and symptom onset (symptoms appeared four days after the second dose), but this single case report cannot establish definitive causation. The simultaneous UC flare remains a potential alternative explanation.

The pathophysiological mechanisms discussed (cytokine dysregulation, IL‑36 overexpression, Th1/Th17 pathways) are derived from the existing literature on paradoxical anti‑TNF reactions, not from mechanistic data generated in this case. These hypotheses are offered as context for clinicians, not as proof of causality.

Our study underscores the necessity for a multidisciplinary approach in managing IBD patients who present with mucocutaneous manifestations such as PPV. We also emphasize the importance of heightened awareness regarding paradoxical reactions in patients undergoing biotherapies like adalimumab.
